# Novel Algorithm for Non-Invasive Assessment of Fibrosis in NAFLD

**DOI:** 10.1371/journal.pone.0062439

**Published:** 2013-04-30

**Authors:** Jan-Peter Sowa, Dominik Heider, Lars Peter Bechmann, Guido Gerken, Daniel Hoffmann, Ali Canbay

**Affiliations:** 1 Department of Gastroenterology and Hepatology, University Hospital, University Duisburg-Essen, Essen, Germany; 2 Department of Bioinformatics, Center for Medical Biotechnology, University Duisburg-Essen, Essen, Germany; Institute of Hepatology, Foundation for Liver Research, United Kingdom

## Abstract

**Introduction:**

Various conditions of liver disease and the downsides of liver biopsy call for a non-invasive option to assess liver fibrosis. A non-invasive score would be especially useful to identify patients with slow advancing fibrotic processes, as in Non-Alcoholic Fatty Liver Disease (NAFLD), which should undergo histological examination for fibrosis.

**Patients/Methods:**

Classic liver serum parameters, hyaluronic acid (HA) and cell death markers of 126 patients undergoing bariatric surgery for morbid obesity were analyzed by machine learning techniques (logistic regression, k-nearest neighbors, linear support vector machines, rule-based systems, decision trees and random forest (RF)). Specificity, sensitivity and accuracy of the evaluated datasets to predict fibrosis were assessed.

**Results:**

None of the single parameters (ALT, AST, M30, M60, HA) did differ significantly between patients with a fibrosis score 1 or 2. However, combining these parameters using RFs reached 79% accuracy in fibrosis prediction with a sensitivity of more than 60% and specificity of 77%. Moreover, RFs identified the cell death markers M30 and M65 as more important for the decision than the classic liver parameters.

**Conclusion:**

On the basis of serum parameters the generation of a fibrosis scoring system seems feasible, even when only marginally fibrotic tissue is available. Prospective evaluation of novel markers, i.e. cell death parameters, should be performed to identify an optimal set of fibrosis predictors.

## Background

Non-alcoholic fatty liver disease (NAFLD) is an increasingly prevalent disease entity, affecting up to one-third of Europeans [1]. NAFLD may progress to non-alcoholic steatohepatitis (NASH) with or without fibrosis and thus predisposes to liver cirrhosis, end stage liver disease and hepatocellular carcinoma (HCC) [2]. Approximately 33% of patients with NASH develop fibrosis and 15% develop cirrhosis [3]. To date, the diagnosis of NASH is established by histological means, including inflammation, steatosis, hepatocellular injury and ballooning. According to Kleiner *et al.*, fibrosis in NAFLD is assessed in a 7-stage system, which includes detailed evaluation of perisinusoidal and periportal fibrosis [4]. However, liver biopsy is associated with a limited, but significant risk of adverse events (bleeding, infection) and a significant rate of observer- and sampling errors, specifically for assessment of fibrosis [5], [6]. Given the high prevalence of NAFLD and the limitations of liver biopsies, biomarkers and surrogate parameters of fibrosis might evolve as important diagnostic means in NAFLD patients.

Hepatocyte cell death in NAFLD is an important predictor of hepatic stellate cell (HSC) activation and thus indirectly of fibrogenesis [7], [8]. Recently, assessment of hepatocyte apoptosis by quantification of soluble cytokeratin 18 (CK-18) has been validated in large cohorts of NAFLD patients as a novel biomarker of disease activity [9]. As engulfment of remnants from apoptotic hepatocytes (apoptotic bodies) by non-parenchymal cells directly and indirectly activates HSCs [10], [11], fibrosis was also found to correlate with serum CK-18 levels in some studies [12], [13]. Another derivate marker with regard to collagen production is hyaluronic acid (HA). HA serum levels increase with progressive fibrosis [14]. In fact, some studies showed a correlation between serum HA and fibrosis stage in chronic liver diseases, including NAFLD [15], [16]. However, in validation studies, individual biomarkers failed to accurately predict fibrosis [17]. Thus, diagnostic multi-panel tests, including a variety of individual parameters have recently been implied as non-invasive fibrosis tests. Most tests have been established in HCV patients and might lack validity in NAFLD patients [18], [19]. Novel tests that include cell death markers, designed for fibrosis assessment in NAFLD, are emerging, but still require large-cohort validation studies [20]. Limitations of these scoring systems comprise small cohorts [21], comparison of no or low-grade cirrhosis *vs.* high-grade of cirrhosis [21], [22], and inclusion of metabolic or biometric parameters increasing the effort needed to generate this score for individual patients [21], [23]. To monitor early fibrogenesis and progression of disease it would be imperative to differentiate even low grades of fibrosis. This is of utmost clinical significance, as pre-existing chronic liver diseases – even in early fibrotic stages – predispose to acute-on-chronic liver failure [24]. However, currently available non-invasive tests for fibrosis fail to distinguish early fibrosis stages.

Here, we aim to introduce a novel score of non-invasive fibrosis parameters, designed specifically for NAFLD. To this end, we used machine-learning algorithms, which are widely used for prediction and classification problems in biomedical research [25]. Typically, a supervised learning strategy is used for training a machine learning algorithm with a set of training samples, for which the input parameters and associated target classes are known. In the setting presented here, these algorithms were trained to differentiate between fibrosis stage 1 and fibrosis stage 2 within a cohort of adipose NAFLD patients, utilizing serum derived parameters.

## Materials and Methods

### Ethics Statement

The study protocol conformed to the ethical guidelines of the 1975 Declaration of Helsinki and was approved by the Institutional Review Board (Ethics Committee) at the University Hospital of Essen. All patients provided written, informed consent before enrollment.

### Patients and Sample Acquisition

126 morbidly obese patients (BMI: 52.2±0.7 kg/m^2^; age: 45.2±0.96; males/females: 28 (22.2%)/98 (77.8%)) who underwent bariatric surgery at a renowned center for bariatric surgery were included (Tab. 1). Some clinical data on this collective have been published previously in a different approach of analysis [26]. Indication for bariatric surgery was made according to NIH guidelines (BMI ≥40 kg/m2 or ≥35 kg/m2, plus co-morbidities). Subjects reporting excessive alcohol consumption (>20 g/day in males or >10 g/day in females) indicating alcoholic liver disease were excluded. Liver serum parameters (ALT, AST) were determined in the central laboratory unit of the University Hospital Essen by standardized methods. Liver biopsies were obtained during the bariatric surgery as wedge biopsy. Individual specimens were stored in 4% formalin solution (Roth, Karlsruhe, Germany) for histological examination. The fibrosis stage was assessed in a blinded fashion by a single pathologist according to Kleiner *et al.* [4].

**Table 1:** Demographic and basic parameters of the investigated patient collective.

Parameter	Fibrosis Stage 1 (1a, 1b, 1c)	Fibrosis Stage 2
Age (y)^1^	45.04±2.54	45.84±0.99
Gender^1^	M 16% (n = 4)/F 84% (n = 21)	M 24% (n = 24)/F 76% (n = 77)
BMI (kg/m^2^)^1^	51.96±1.59	52.25±0.81
AST^1^	27.12±2.65	29.98±1.61
ALT^1^	34.44±4.81	37.67±2.59
gamma-GT^1^	34.24±4.48	55.29±12.40
LDH	232.92±9.99	226.47±5.31
bilirubin	0.56±0.08	0.55±0.03

No significant differences were found between stage 1 and stage 2.

1 These data have been previously shown in a different analysis of this patient cohort in Kälsch *et al.* [28].

### ELISA

Sera were collected upon admission and throughout hospitalization and stored within 2 h at −20°C until testing. Individual values of clinical and standard laboratory data, overall cell death and apoptosis markers M65 and M30 (determined by the M30-Apoptosense and M65 ELISAs from Peviva; Bromma, Sweden), as well as hyaluronic acid (Corgenix, Bloomfield, CO, USA) were determined. All procedures were conducted according to the manufacturers’ instructions.

### Dataset and Statistics

The resulting parameters used for classification were ALT, AST, M30, M65 and HA. Samples with incomplete parameters were discarded prior to analysis. The final dataset consisted of 25 samples of fibrosis status 1 (including stages 1a, 1b, 1c; handled as positive samples) and 101 samples of fibrosis status 2 (representing negative samples). We compared all parameters of fibrosis-status 1 (1a, 1b, 1c) with the corresponding parameters of fibrosis-status 2 using Wilcoxon Signed-Rank tests.

### Machine Learning

Several machine learning techniques were evaluated, namely logistic regression (logReg), k-nearest neighbors (knn), linear support vector machines (SVM), rule-based systems (RB), decision trees (DT) and random forest (RF). For the logistic regression, we used the implementation in the *stats* package of R (http://www.r-project.org) with standard settings. The k-nearest neighbor implementation in the R package *class* was used with k = 3. The SVM implementation in the package *kernlab* of R was used with the *vanilladot* kernel. For the rule-based systems we used the *Part* [27] implementation provided in the R package *RWeka*. For the DTs we used the implementation in the *rpart* package and for the RFs [28] we used the implementation in the *randomForest* package of R. In our application each RF consisted of 2000 randomly and independently grown decision trees. When using the trained RF for prediction, an unseen instance was assigned to the positive class voted for by at least 50% of the trees. The importance of each variable for the correct classification can be assessed by determining the decrease in Gini impurity [29].

### Cross-validation

All machine learning methods were validated using ten-fold leave-one-out cross-validation [29] to assess for the different machine learning methods the mean prediction sensitivity, specificity, and accuracy (see formulas below) and the ability to generalize to unseen instances.

For each test in the cross-validation, the sensitivity (SN), specificity (SP), and accuracy (AC) were calculated according to:
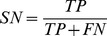


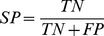


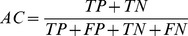
with true positives TP, false positives FP, false negatives FN and true negatives TN. Furthermore, we calculated the Receiver Operating Characteristics (ROC) curve [30] and the corresponding area under the curve (AUC) with ROCR [31]. The ROC curve is built by plotting sensitivity *vs.* specificity for every possible cut-off between the two classes.

### Permutation Test

All machine learning methods were tested for significance using a permutation test. The AUC distribution for each classifier was calculated by ten-fold leave-one-out cross-validation. 1000 ( = N) random permutations of the class labels were generated and the classifiers were trained and evaluated again. Each of the resulting AUC distributions of the permutation was compared with the *real* AUC distribution using Wilcoxon Signed-Rank test. The number k of permutations for which the mean AUC had no significant differences compared to the *real* AUC was counted for each classifier. The p-value of the permutation test was calculated by
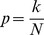



### Statistical Comparison

All models were compared by applying Wilcoxon Signed-Rank test on the AUC distributions from the ten-fold leave-one-out cross-validation runs. The null hypothesis was that there are no differences between the compared classifiers.

## Results

### Patient Characteristics

Detailed data of the included patients can be found in table 1, comprising distribution of demographic parameters as well as standard parameters of liver damage (transaminases, bilirubin, gamma-GT and LDH). Serum parameters for liver damage were within normal range and no pathological alterations were detected. As the patient collective consisted of adipose patients, the BMI was significantly above normal ranges. Partial data of this patient cohort has been presented before in a different type of analysis in Kälsch *et al.* [26].

### No Prognostic Value of Individual Parameters

We found no significant differences between ALT, AST, M30, M65 and HA of fibrosis status 1 and 2 with regard to Wilcoxon Signed-Rank test (p = 0.55, p = 0.30, p = 0.70, p = 0.87 and p = 0.86, respectively).

### Accuracy of the New Diagnostic Algorithm for Prediction of Fibrosis Stage

**Figure 1:**
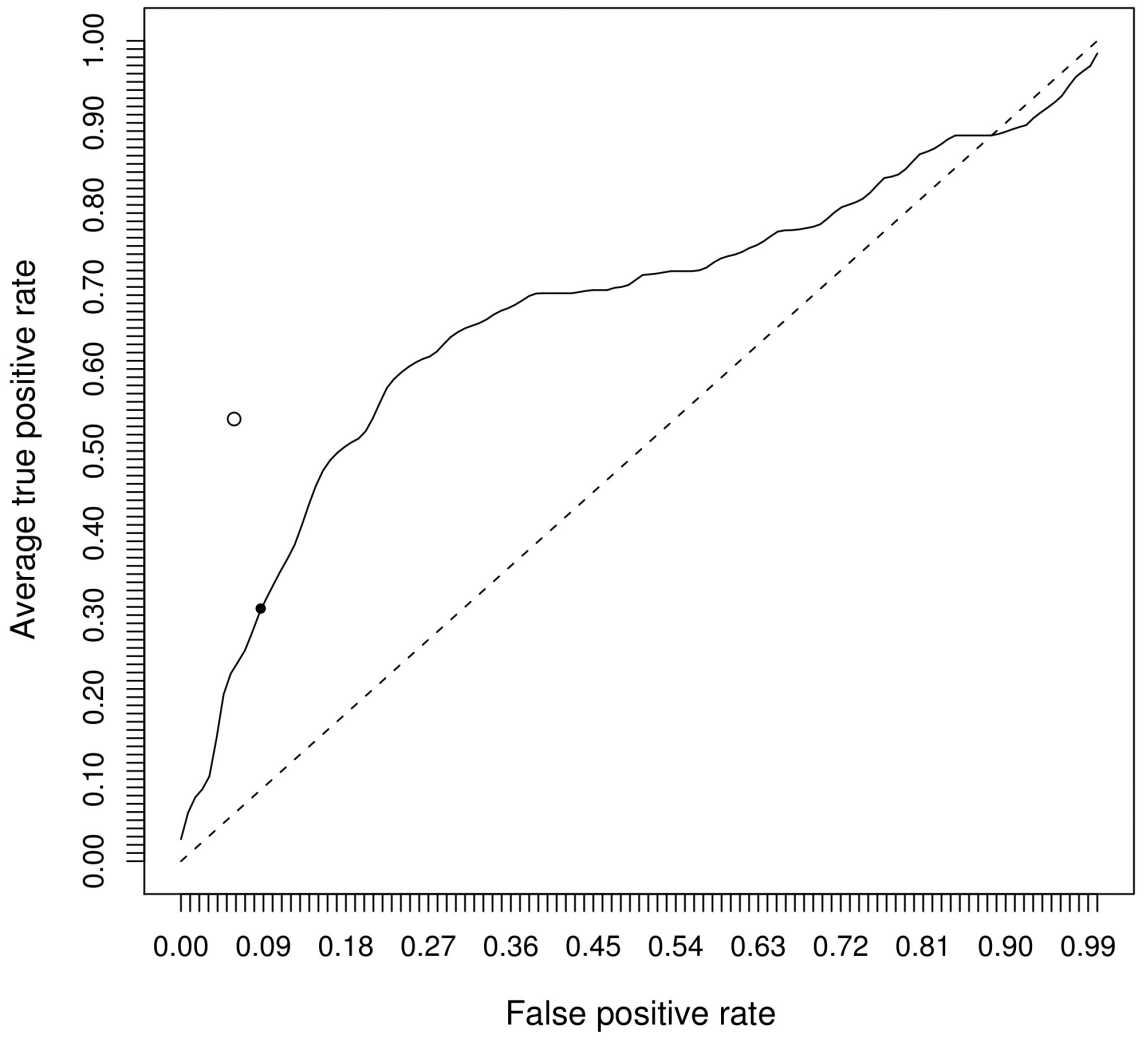
Prediction performance. The ROC curve of the RF is shown (p = 0.008). Black dot: performance of the 3 nn (p = 0.02); white circle: performance of the DT (p value = 0.099). The dashed line marks the performance by chance.

Most of the classifiers were not able to accurately predict the fibrosis status (or the results were insignificant according to the permutation test). Neither the logistic regression, nor support vector machines, nor rule-based systems were able to predict the fibrosis status from the given blood parameters. However, random forests and the k-nearest-neighbor algorithms had an accuracy of about 79%. The RFs reached an AUC of 0.6704+/−0.0062 (p = 0.008). A cutoff of 0.22 between positive and negative samples led to a sensitivity of over 60% and a specificity of 77% (Figure 1). The knn reached a sensitivity of 30.8% with a specificity of 91.3% (p = 0.02), which is slightly worse compared to the RFs (sensitivity of 30.8% with specificity of 92.2%). Decision trees displayed the best performance (sensitivity of 53.9% and specificity of 94.2), but failed to reach a significance level of 5% in the permutation test (p = 0.099).

**Figure 2:**
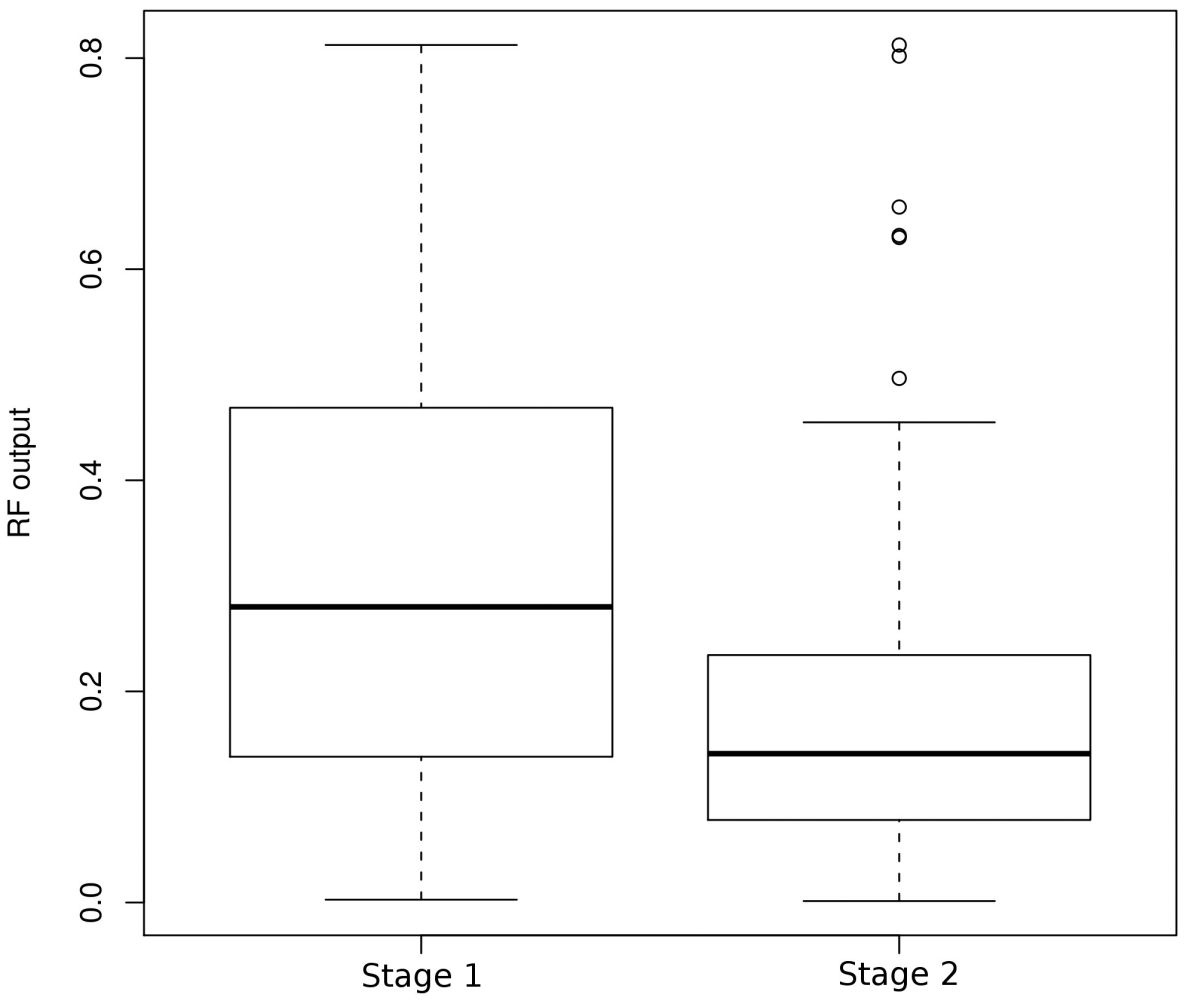
Boxplots of the outputs of the RFs. On the y-axis the predicted class probabilities for stage 1 and stage 2 are plotted. Generally, the RFs give higher prediction values for subjects from stage 1 compared to subjects from stage 2. The upper and the lower quartiles, the median (bold line) as well as outliers (circles) are shown.

Furthermore, we analyzed the prediction output for each sample. The output of the RFs is shown as boxplots for each of the two classes in Figure 2. Subjects from stage 1 had higher prediction values compared to subjects from stage 2 on average.

**Figure 3:**
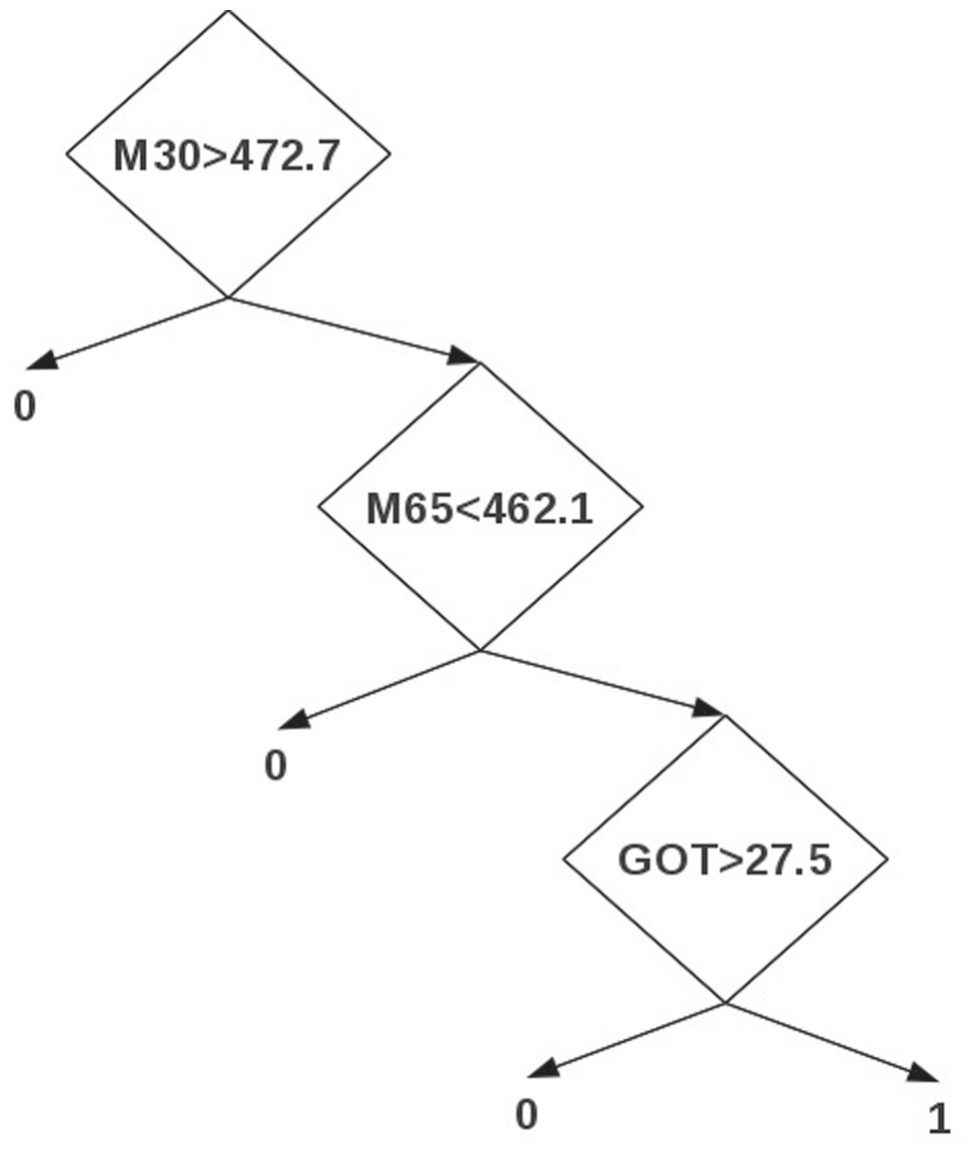
Decision Tree. The decision tree (DT) focuses on the parameters M30, M65 and AST (GOT), which is in partial agreement with the RFs. At the first level, the DT focuses on M30. If the M30 value for a given sample is less than 472.7, it is assigned to the negative class (0). If the M30 value is greater than 472.7, it is transferred to the next level that uses M65. If the M65 value for the given sample is greater than 462.1, it is assigned to the negative class, otherwise it is transferred to the last level. The last level in the DT focuses on GOT. If the GOT value for the given sample is less than 27.5, the sample is assigned as negative, otherwise as a positive sample (1).

As mentioned before, RFs are able to identify the most important parameters for the classification process. The RFs identified M30 and M65 as being slightly more important than the other variables, which is in accordance with the DTs (only M30, M65 and AST are used in the trees, see Figure 3). The estimated importance for each parameter is shown in table 2.

**Table 2:** Importance analysis.

parameter	importance
ALT	6.76
AST	6.53
M30	9.90
M65	9.57
hyaluronic acid	8.28

## Discussion

Diagnosis of liver fibrosis or cirrhosis provides important clinical information. Different etiologies and conditions, affecting liver integrity and function may increase susceptibility to various toxins (e.g. drugs) or viruses and are associated with enhanced morbidity or mortality in acute liver injury [24]. Pre-existing liver disease, though, often persists in presence of marginally or only slightly elevated classic liver parameters, which would not suggest a liver biopsy to test for fibrosis. This is particularly common in obese NAFLD patients, as we have recently shown. Then again even a liver biopsy – comprising only 1/50.000^th^ of the total liver volume – may not lead to an unambiguous judgement by a pathologist [21], [32], [33]. Moreover, liver biopsy is a painful invasive technique, which also confers a certain health risk, especially for patients with already reduced functional liver mass. Thus non-invasive methods that indicate progression to fibrosis or cirrhosis are needed that more reliably predict which patients should undergo a liver biopsy, or have their fibrosis status estimated if a biopsy is clinically not feasible.

A major concern for all attempts to establish such adequate scoring systems, is that as reference the gold standard has to be applied, which is biopsy and subsequent histological examination. As mentioned above, this technique contains two main sources of error that have to be considered when calculating a score for fibrosis/cirrhosis estimation [32], [33].

Previous studies could clearly demonstrate that single serum parameters are not sufficient to reach the sensitivity or specificity of liver histology [19], [21]. A scoring system combining different parameters seems to be the most promising approach. Knowing that cell death by apoptosis is a critical step for NASH development, integrating markers of cell death appears reasonable. Subsequent activation of HSC by hepatocyte apoptosis leads to ECM production [11], [34]. In our calculations we included an importance analysis, demonstrating the highest importance for cell death parameters and hyaluronic acid, which has been linked to cirrhosis and collagen production [14]. This would fit with current theories discussing hepatocellular death and subsequent activation of non-parenchymal cells as crucial events in fibrogenesis and progression to cirrhosis in chronic, but also in acute liver injury [35].

While there have been previous attempts to establish a non-invasive method to detect fibrosis or cirrhosis, most groups have done their analyses in patients with viral hepatitis, where abundant collagen deposition and progression to cirrhosis is given. Systems which were established in NAFL/NASH cohorts often distinguish only no or low-grade fibrosis from cirrhosis [22]. This obviously does not suffice to monitor fibrosis progression in NAFLD, where progression is rather slow compared to viral or alcoholic hepatitis. Moreover, when at some point a therapeutic intervention against fibrosis is possible, detecting established cirrhosis vs. low fibrosis may not be helpful to identify patients in need of this – hypothetical – medication. Thus, early detection of fibrosis may be crucial for a therapeutic approach to counter fibrosis progression, lowering vulnerability to additional, acute injury. A recent study by Tomeno *et al.* assessed the efficacy of real time transient elastography to detect liver fibrosis [36]. While the calculated liver fibrosis index correlated well with histological scores in chronic hepatitis, no such correlation was found for NAFLD. One could speculate that either overweight or fat deposition within the liver may interfere with elastography measurements in NAFLD. In any case, elastography has to be interpreted with care in the setting of NAFLD. Some NAFLD related fibrosis scores took the distinctiveness of this etiology into account and included biometric data [21], [23], which requires additional data for each patient (height, weight). This may not be a limitation for a few individual patients, though it has to be viewed in the light of increasing numbers of overweight or obese individuals in the general population. Constraining the utilized markers to serum derived parameters reduces the clinical course of action to a single blood withdrawal. This would allow a true screening of patients without the need to take additional measurements.

Another major difference to other scoring systems is the employment of non-linear machine learning techniques. For instance, the RFs allow to estimate variable importance and hence can be used to further improve prediction performance. Moreover, some of these methods provide simple rules, which can be applied in clinical settings to predict the fibrosis status of new patients and thus estimate a potential progression of the liver disease.

Limitations of our results are due to the number of patients with a complete dataset and moreover on the limited variability of fibrosis stages. As we did our investigation on a mostly obese collective with a probable NAFLD/NASH type of liver damage, the generation of fibrotic tissue is expected to be rather slow, in contrast to viral etiologies or alcohol abuse. Thus, we decided to differentiate between the two lowest fibrosis stages and trained the models accordingly. Although the dataset has the mentioned limitations, we were able to build a model that is able to discriminate between the two fibrosis classes with a reasonable sensitivity and specificity. Prospective studies recruiting a more diverse group of patients, exhibiting the full range of fibrotic stages (0–4) could increase the quality of the prediction model. Moreover, a broader range of parameters should be included to identify those parameters with the highest importance for diagnosis of fibrosis using machine learning techniques. Another possible option is to combine serum parameters with other non-invasive data, e.g. transient elastography of the liver. Additionally, it might also be possible to accurately predict the NAS status with an enlarged dataset.
